# Lassa Virus—A Persistent and Evolving Threat

**DOI:** 10.3390/microorganisms14092047

**Published:** 2026-09-14

**Authors:** Meng Hu, Emily Mantlo, Cheng Huang

**Affiliations:** 1 Department of Pathology and Galveston National Laboratory, University of Texas Medical Branch, Galveston, TX 77550, USA; menhu@utmb.edu; 2 Department of Microbiology and Immunology, Global Health Institute, Upstate Medical University, Syracuse, NY 13210, USA; mantloe@upstate.edu

**Keywords:** Lassa fever, LASV, geographic distribution, infection, transmission, pathogenicity, pathogenesis, medical countermeasures

## Abstract

Lassa virus (LASV), the most important human pathogen within the *Arenaviridae* family, causes Lassa fever (LF), an acute hemorrhagic fever disease endemic in West Africa with high case fatality rates, particularly in pregnant women. Currently, there are no licensed vaccines or antivirals approved for clinical use against LASV infection. Despite its significant impact on public health, important knowledge gaps remain regarding LASV disease burden, transmission dynamics, pathogenesis, and virus−host interactions, impeding effective disease control and development of therapeutic interventions. The emergence of new lineages and the remarkable genetic diversity of LASV poses substantial challenges to accurate and prompt diagnosis, epidemiological surveillance, and the development of broad-spectrum medical countermeasures. Here, we synthesize current knowledge on the disease burden of LF, recent outbreaks, the geographic distribution and genetic diversity of LASV, infection and transmission in natural reservoirs and other susceptible hosts, emerging evidence suggesting possible sexual transmission, viral pathogenesis, host immune responses, diagnostic advances and challenges, and ongoing preclinical and clinical efforts to develop effective vaccines and antivirals.

## 1. Background

Lassa virus (LASV) is the causative agent of Lassa fever (LF), a severe and often fatal zoonotic disease endemic in West Africa [1,2,3]. LASV belongs to the genus *Mammarenavirus*, family *Arenaviridae*, order *Hareavirales*, and *class Bunyaviricetes* [4]. Symptomatic LASV infection in humans typically begins with nonspecific febrile, “flu-like” manifestations, including fever, headache, sore throat, cough, myalgia, nausea, vomiting, and diarrhea. In severe cases, patients may progress to pulmonary edema, facial edema, acute respiratory distress, coma, seizures, terminal shock, as well as hemorrhagic manifestations (i.e., mucosal or gastrointestinal bleeding) [2,3,5]. Patients usually succumb due to multi-organ failure. Survivors frequently experience long-term sequelae, including sensorineural hearing loss (SNHL), polyserositis, vision disturbances, vertigo, and back pain [6]. The overall LF fatality rate is approximately 1%, and could be 15% or even higher in clinical cases during outbreaks [5]. For example, during the 2015–2016 outbreak in Nigeria, a retrospective study reported a case fatality rate of ~60% in 47 hospitalized patients with confirmed LF [7]. A prospective cohort study (LASCOPE) conducted in 2018 and 2020 reported a 12% case fatality rate in 510 hospitalized LF patients and found that 57% of patients had malaria co-infection at the time of admission [8]. Infection is particularly devastating in pregnant women, resulting in exceptionally high fetal mortality (>75%) and substantial maternal mortality of up to ~50%, especially during the last month of gestation [9]. There is a critically unmet need for effective medical countermeasures against LASV infection. Accordingly, LASV has been designated as a priority pathogen by the World Health Organization (WHO) [10]. The virus is classified as a Select Agent in USA [11]. Experimental studies involving infectious LASV are limited by the requirement of biosafety level 4 (BSL-4) facilities [12].

A literature search was initially conducted by using the Advanced Search function in the PubMed database and included studies published up to the end of March 2026. Additional relevant publications published through August 2026 were then identified and incorporated. The search strategy used the terms “Lassa virus” and “Lassa fever” in the “All Fields” category. The publication date ranged from 1970 to 2026. We primarily focused on original research articles reporting representative findings and directed readers to review articles that provide broader and more in-depth coverage on specific topics. Of the resulting 1481 articles published in English, 139 reports were selected to cover the key topics, including LASV geographic distribution, prevalent virus lineages, infection and transmission in natural reservoirs and other susceptible hosts, pathogenesis, host immune responses, and ongoing efforts to develop effective medical countermeasures. In addition, relevant reports and sources from U.S. Centers for Disease Control and Prevention (CDC), WHO, National Institute of Allergy and Infectious Diseases (NIAID), ClinicalTrials.gov, or Nigeria Centre for Disease Control and Prevention (NCDC) were included. The added value of this review lies in the synthesis of the most recent literature available through the end of August 2026, covering advances in LF epidemiology and diagnostics, as well as progress in the preclinical and clinical development of vaccine and antiviral candidates. Furthermore, we presented a curated dataset of the sequences of human LASV isolates, providing a resource for investigators interested in LASV molecular epidemiology, evolution, and vaccine design. It should be noted that this review is narrative rather than systematic.

## 2. Geographic Distribution, Infection, and Transmission

LASV is the most prevalent hemorrhagic fever-causing arenavirus in humans. From 2018 to 2025, NCDC reported a yearly average of 7105 suspected LF cases with 995 confirmed LF cases and 192 deaths (Table 1) [13]. However, the actual burden of human infections is substantially higher due to limited diagnostic capacity, lack of clinical awareness, and a high proportion (~80%) of asymptomatic and mild infections.

An early study in 1987 estimated that approximately 100,000–300,000 LASV infections occur in humans annually in West Africa, resulting in 5000 deaths [14]. A recent study using mathematic modeling and machine learning predicted 897,700–4,383,600 human infections each year across West Africa [15]. From 2020 to 2023, the Enable Lassa Fever Research Programme has conducted a multi-country, community-based prospective cohort study involving more than 20,000 participants across Benin, Liberia, Nigeria, and Sierra Leone (Enable 1.0 Lassa Research Study) [16]. The Enable 1.0 study has reported an overall LF incidence rate of 1.27 per 1000 person-years in endemic countries and, for the first time, documented a higher risk of LF infection in children aged 2–17 years than in adults (adjusted incidence rate ratio: 1·96; 95% CI: 1·04–3·72). Marien et al. (2025) conducted serosurveillance in Faranah, Upper Guinea, and found extremely high LASV IgG seroprevalence (>80%) in adults and over 50% seropositivity in children by 5 years of age [17]. In a large retrospective study of 153 pediatric patients with neurological findings and suspected or confirmed LF, LASV RNA was detected in 27% of cerebrospinal fluid samples tested [18,19]. This survey suggests LASV infection in the central nervous system (CNS) may occur frequently among pediatric LF patients presenting with neurological signs and symptoms. Despite the high incidence of infections, reported acute LF cases remain low, suggesting substantial under-recognition and frequent asymptomatic or mild infections. Taken together, these surveillance data highlight the persistent and largely hidden burden of this neglected infectious disease and emphasize the need for continued efforts to define the true burden of LF, which is key to public health management and disease control.

**Figure 1:**
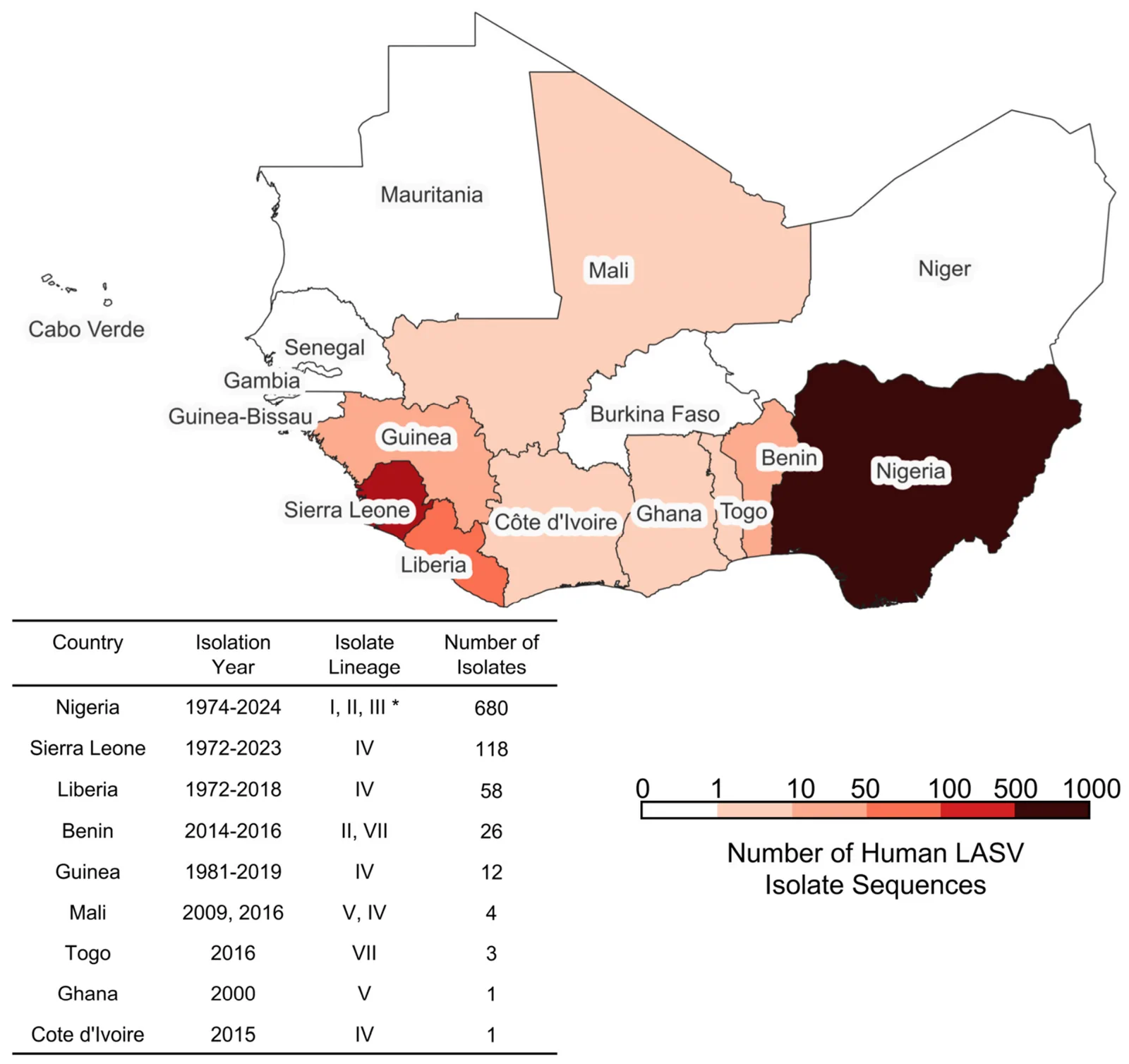
Analysis of human LASV isolates with viral genomic RNA sequence deposited in National Center for Biotechnology Information GenBank (1974–2024). Human LASV sequence data (1974–2024) deposited in the GenBank database were retrieved from the Lassa dataset in Nextstrain (https://nextstrain.org/) and analyzed for geographic distribution and lineage (Supplementary Materials). Duplicated strains originating from multiple sources and strains with unknown host information were excluded. The data represent sequence availability and the lineage information in different countries, rather than disease incidence, prevalence, lineage abundance, or geographic risk. * Among the isolates from Nigeria, one isolate isolated in 2001 was classified within lineage IV.

LASV exhibits remarkable genetic diversity among isolates and has been classified into seven major lineages (I−VII) (Figure 1). LASV genomic RNA sequence can differ by up to 32% in the L genomic RNA and 25% in the S genomic RNA among LASV strains [2]. The high sequence diversity of LASV poses a substantial obstacle to the development of effective and broad-spectrum diagnostic tests and countermeasures. The distribution of LASV lineages is often associated with certain geographic regions, reflecting the path of viral evolution and transmission.

We retrieved publicly available human LASV sequence data collected between 1974 and 2024 from the Nextstrain Lassa dataset (https://nextstrain.org/) [20] and analyzed their geographic distribution. The Nextstrain platform collects viral genome sequences deposited into the National Center for Biotechnology Information (NCBI) GenBank database and tracks the real-time evolution and spread of viral pathogens of high public health importance, including LASV [21]. We included three isolates identified by our group during the 2022–2024 LF outbreaks in Nigeria. The corresponding GenBank accession numbers are provided in Supplementary Materials. The dataset was restricted to isolates with host “Homo sapiens” or “Human”. We excluded duplicated isolates originating from multiple sources, as well as isolates lacking host or geographic information. Isolates containing either full-length or partial sequences of the viral L or S segments were included. Viral lineages were assigned based on classifications available in Nextstrain or as reported in original publications associated with each isolate. These publications were identified via GenBank accession numbers and retrieved from PubMed where available. Isolates for which lineage information could not be established were categorized as “data not reported”. It should be noted that the resulting data represent sequence availability and lineage information in different countries, rather than disease incidence, prevalence, lineage abundance, or geographic risk. Countries with stronger sequencing capacity inevitably appear to have more isolates.

In West Africa, Nigeria reported 680 human LASV isolates from 1974 to 2024, with most isolates belonging to lineages I, II, and III (Figure 1). Sierra Leone accounted for 118 human LASV isolates (lineage IV) from 1972 to 2023. Liberia reported 58 human LASV isolates (lineage IV) from 1972 to 2018. Benin reported 26 human LASV isolates (lineage II or VII) from 2014 to 2016 [22], and Guinea reported 12 human LASV isolates (lineage IV) from 1981 to 2016. Mali, Togo, Ghana, and Côte d’Ivoire have reported fewer than five human LASV isolates, representing lineages IV, V, and VII, associated with sporadic LF cases [23] (Figure 1). Lineage VI has thus far been isolated exclusively from rodents [24]. Notably, there are increasing reports on human and rodent infection in other West African countries and in travelers, raising concerns that the geographic range of LASV is expanding [25,26,27].

The primary natural reservoir of LASV is the peri-domestic rodent *Mastomys natalensis* [1,2]. Although *M. natalensis* is widely distributed across sub-Saharan Africa, LASV has only been documented in *M. natalensis* populations in West Africa [28]. In endemic regions, up to 30% of *Mastomys* rodents may carry LASV [14]. LASV infection in *Mastomys* rodents could be persistent as indicated in an early study [29]. More recent studies demonstrated that lab-reared adult *M. natalensis* are highly susceptible to LASV isolates of diverse geographic origins via various infection routes, including intraperitoneal, intramuscular, subcutaneous, intranasal, and oral exposure [30,31]. Adult *M. natalensis* typically developed systemic infection without overt disease signs, with viral RNAs detected in multiple organs, including kidney, lymph nodes, spleens, lungs, liver, reproductive organs, brain, gastrointestinal tract, and heart [30]. The infection outcome of LASV in *M. natalensis* seemed to be age-dependent [32]. Mice younger than two weeks old established persistent infections lasting up to 16 months, whereas mice older than four weeks old had transient infections [32]. Horizontal transmission studies demonstrated seroconversion in approximately 40% of contact-exposed and 25% of fomite-exposed adult *M. natalensis* [30]. Persistently infected *M. natalensis* can transmit LASV to their offspring at rates exceeding 99% from female and ~17% from male animals [32]. Even transiently infected mice can transmit LASV to naïve mice [32]. In addition to *M. natalensis,* other rodent species have been identified as LASV natural reservoirs, including *M. erythroleucus*, the host of LASV lineage III and IV isolates, and *Hylomyscus pamfi*, the reservoir of the lineage VI Kako strain [33].

**Figure 2:**
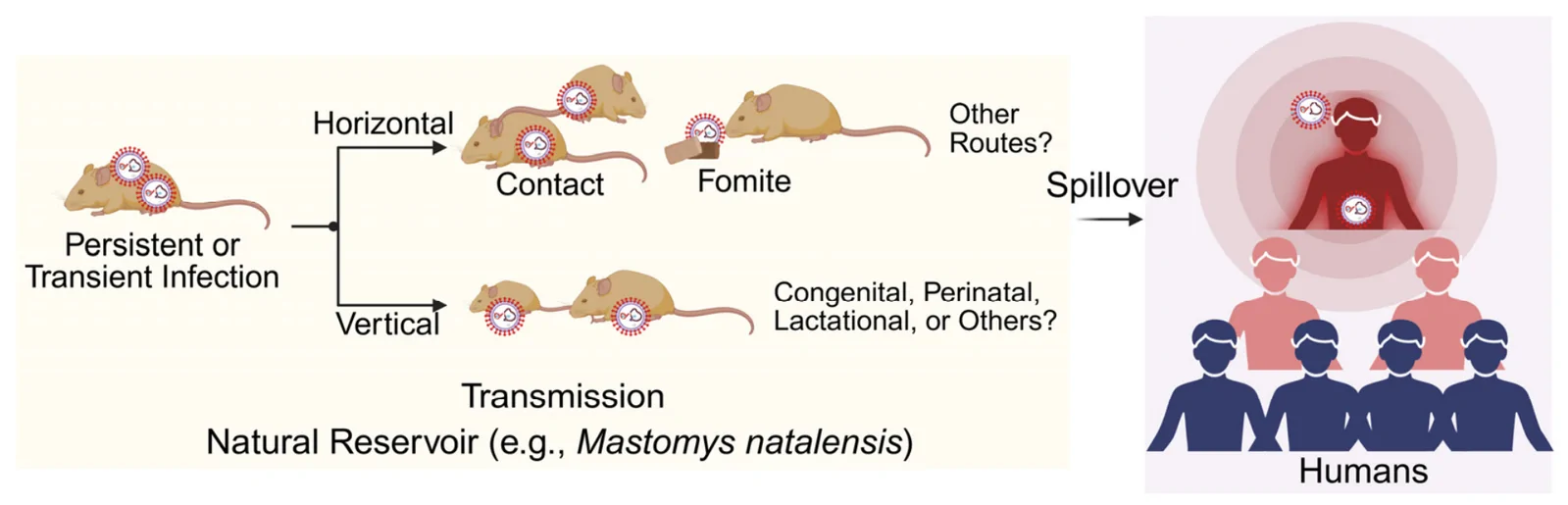
Schematic overview of LASV infection and transmission in nature. LASV is maintained in its primary rodent reservoir, *M. natalensis*. Virus infection in *M. natalensis* could be either persistent or transient. Infected animals may spread the virus horizontally to naïve animals via contact (direct), fomite (indirect), or potentially other routes (e.g., sexual transmission), or to their offspring possibly involving congenital, perinatal, lactational, or close-contact mechanisms. Spillovers to humans can occasionally occur through inhalation of aerosols of urine or feces from infected rodents, ingestion of contaminated food and/or water, or direct contact. Human-to-human transmission of LASV can occur in clinical or household settings through direct contact with blood and other body fluids, contaminated materials, and infection-control failures.

LASV occasionally spills over from rodent reservoirs to humans, causing LF disease [34] (Figure 2). To understand the pathogenesis and transmission of LASV, several relevant animal models have been established, including murine, guinea pig, and non-human primate (NHP) models (reviewed in [35]). As described above, in the rodent models, LASV can transmit horizontally, through exposure to infectious oropharyngeal secretions, urine, and feces [32]. In NHPs, severe disease is often associated with systemic virus dissemination and inflammatory responses, especially in late stages [36].

Although LF is believed to be endemic in West Africa for centuries, LASV was identified in humans for the first time in 1969 in Nigeria [37,38]. Spillovers to humans result from inhalation of aerosols of urine or feces from infected rodents, ingestion of contaminated food and/or water, or direct contact. Human-to-human transmission is uncommon and occurs mainly in healthcare or household settings, likely via contact with blood and other bodily fluids, contaminated materials, and infection-control failures [39,40,41]. Recently, accumulating evidence has suggested that sexual transmission may represent an additional route for LASV infection. McElroy A et al. reported the detection of LASV RNA in the semen of a patient with acute infection at 15, 20, and 48 days after symptom onset [42]. Thielebein A et al. detected moderate to high levels of LASV RNA in the semen of survivors for up to one year after hospital discharge [43]. Notably, infectious LASV was isolated from semen samples up to nine months after discharge even though many of the patients were treated with ribavirin. These findings raised concerns about the potential risk of sexual transmission of LASV by convalescent patients, including those who have received ribavirin treatment [43]. However, human sexual transmission remains suspected or biologically plausible but has not been established. In line with the clinical findings, high levels of infectious LASV were detected in experimentally infected *M. natalensis* in sperms and reproductive organs (i.e., testes, seminal glands, prostate, and epididymides) [31,32,44]. Moreover, intravaginal inoculation of LASV resulted in a systemic and productive infection in female *M. natalensis* [44]. However, it remains to be determined whether sexual transmission naturally occurs in *M. natalensis*. In guinea pigs, LASV RNA and antigen were detected in multiple reproductive tissues and cell types [45]. These clinical and animal studies collectively suggest that sexual transmission may represent a possible route of LASV spread in humans, which warrants further investigation. The resulting data may inform LF surveillance and disease control, particularly considering the possibility of asymptomatic virus shedding and transmission in the convalescence stage of LF.

## 3. LASV Replication

**Figure 3:**
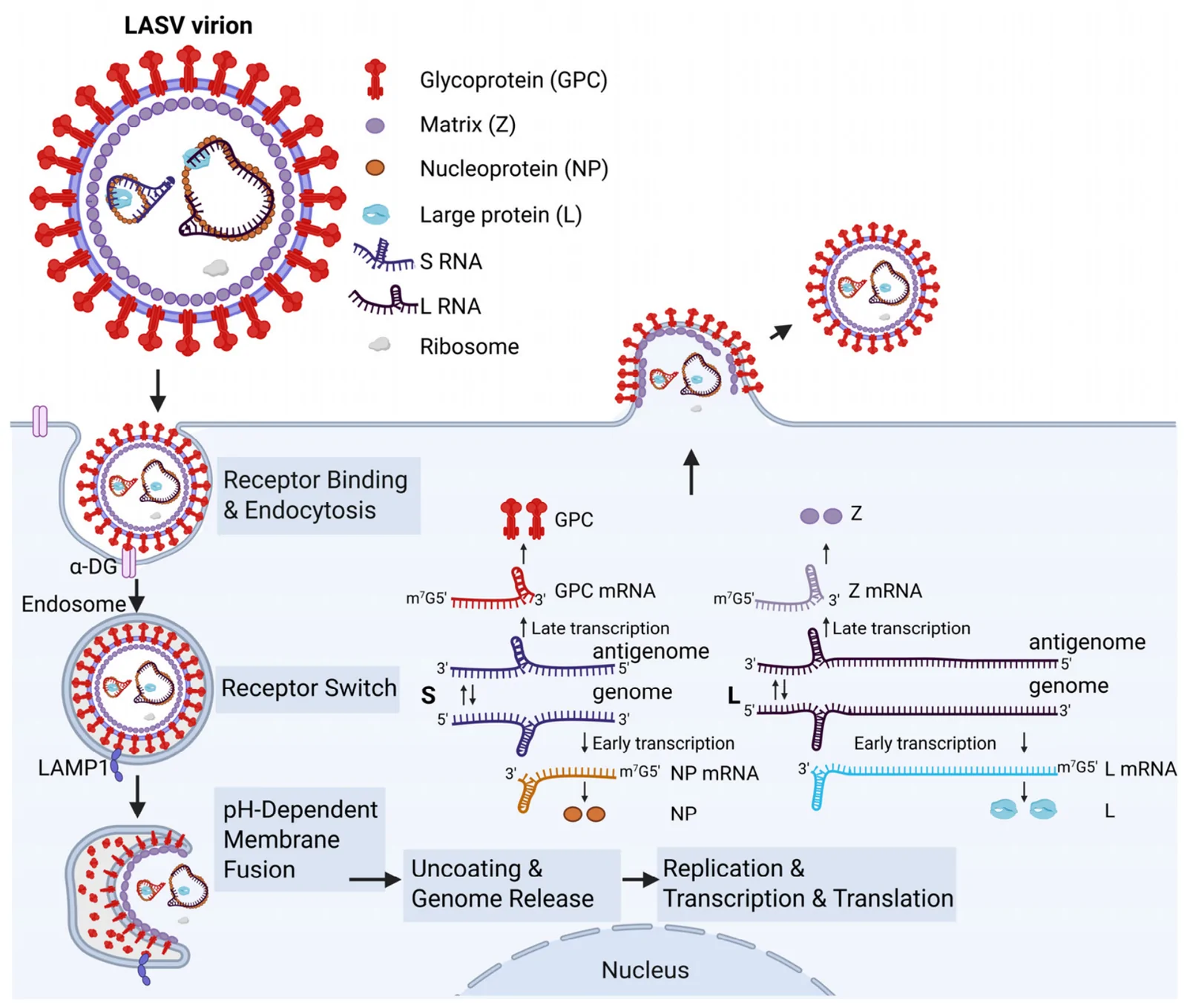
LASV replication cycle. Viral entry is initiated by glycoprotein binding to the cellular receptor α-DG on the host cell surface, followed by endocytosis via macropinocytosis. The low-pH-mediated structural changes in viral GP1 lead to a receptor change to LAMP1. After membrane fusion with the late endosome, viral genomes are released into the cytoplasm, where viral RNA replication, transcription, and viral protein translation occur. The viral glycoprotein precursor (GPC) is synthesized in the endoplasmic reticulum and subsequently transported through the Golgi apparatus to the plasma membrane. Newly synthesized viral genome RNAs and proteins are assembled at the plasma membrane, where progeny virions bud from infected cells. α-DG—alpha-dystroglycan; LAMP1—lysosomal-associated membrane protein 1.

LASV is a negative-sense RNA virus [46]. The virions are enveloped particles ranging from 80 to 200 nm in diameter. The genome consists of two single-stranded RNA segments: a small RNA (S, ~3.5 kb) encoding the glycoprotein precursor complex (GPC) and the nucleoprotein (NP), and a large RNA (L, ~7.2 kb) encoding the small RING finger matrix protein (Z) and the large protein (L) (Figure 3). The terminal regions of both RNA segments are complementary, enabling the formation of panhandle structures of approximately 19–33 nucleotides [46,47].

The GPC (~75 kDa) precursor is processed by host proteases to produce a stable signal peptide (SSP, 58 amino acids), the glycoprotein 1 subunit (GP1, 40–46 kDa), and the glycoprotein 2 subunit (GP2, ~35 kDa), which together form a trimeric complex on the virion surface mediating virus attachment to the host cell and membrane fusion [48] (Figure 3). The L protein (~250 kDa) is the viral RNA-dependent RNA polymerase (RdRp), catalyzing viral RNA transcription and replication. The NP (~63 kDa) encapsulates viral genomic and antigenomic RNAs to form the ribonucleoprotein complex (RNP) along with L [46,49]. The matrix protein Z (~11 kDa), which lines the inner surface of the viral envelope, mediates virion assembly and budding [50]. It also regulates viral RNA synthesis in the late stage of virus replication and contributes to viral suppression of host innate immune responses [51].

LASV replication occurs entirely within the cytoplasm of the host cell. During entry, LASV GP1 binds to the cellular receptor alpha-dystroglycan (α-DG), a highly glycosylated cell-surface protein [52]. After receptor binding, the virion is internalized via macropinocytosis and enters the endosome compartment [53]. The low pH in the endosome triggers the conformational change in GP1, leading to its disassociation from the primary receptor α-DG and switching binding to the endosomal receptor, lysosomal-associated membrane protein 1 (LAMP1) [54,55] (Figure 3). Then, GP2 undergoes conformational rearrangement and subsequently drives the fusion of viral and endosomal membranes [50,56,57]. Compared with influenza viruses, which typically undergo membrane fusion between pH 5.0 to 5.9 [58], LASV GP-mediated membrane fusion can occur at a lower pH between pH 4.0 and 5.0 [59,60].

After membrane fusion, viral genomic RNAs are released into the cytoplasm, where viral RNA transcription and replication occur (reviewed in [61]). Each LASV genomic RNA encodes two open reading frames (ORFs) in opposite orientation separated by a highly structured intergenic region (IGR) that is the bona fide transcription termination signal. During viral mRNA synthesis, the LASV L RdRp cleaves host mRNAs at several nucleotides downstream of the 5′ cap structure and uses the RNA fragments as primers for transcription. Early in infection, NP and L mRNAs are synthesized from incoming genomic RNAs (Figure 3) and direct NP and L protein synthesis. Later, expressed NP and L proteins start to replicate viral genomic RNAs to generate complementary antigenomic RNAs. The antigenomes serve as the template for transcription of viral Z and GPC mRNAs as well as the template for synthesis of progeny viral genomic RNAs (Figure 3). Synthesized viral genomic RNAs and proteins are transported to the plasma membrane, where Z protein drives the assembly and the budding of progeny virus particles from the surface of infected cells (Figure 3) [62,63].

## 4. LASV Pathogenicity and Pathogenesis

Despite decades of research, our understanding of LASV pathogenesis and pathogenicity in humans remains limited. In 1982, Walker et al. examined fatal LF cases and found widely disseminated viral infection in the liver, lung, spleen, kidney, heart, placenta, and mammary gland [64]. The most consistent lesions were hepatic, adrenal, and splenic necrosis. However, none of these lesions were severe enough to explain the cause of death. In 1985, McCormick et al. further studied postmortem liver biopsies from 19 patients with fatal LF [65]. They found that the degree of hepatic damage was not sufficient to implicate hepatic failure and concluded that the hepatitis of LF in humans was not the primary cause of death. In 2022, Shieh et al. examined archived autopsy tissues from fatal cases and detected profound LASV infection across diverse cell types (e.g., endothelial cells, macrophages, and dendritic cells) and tissues (e.g., liver, mucosal tissues, and reproductive and endocrinologic tissues) [66]. Multifocal hepatocellular necrosis with eosinophilic Councilman bodies was observed and the lesions in the liver were often associated with minimal to mild mononuclear inflammatory cell infiltrate. Sporadic necrosis in the spleen, lymph nodes, and adrenal cortex was observed. Other organs exhibited only nonspecific changes or lacked significant histopathologic lesions [66]. In general, the evidence from limited histopathology studies conducted so far indicated that the extent of organ damage is insufficient to explain the cause of death in LF patients.

LASV pathogenicity is affected by multiple factors, including viral factors, virus strains, host factors, and immune responses. In LF patients, a high level of viremia was found to be associated with poor disease outcomes [67]. However, whether severe or fatal disease is primarily driven by unchecked virus replication, dysregulated host response, or both is largely unresolved. Clinical studies demonstrated that severe and fatal LF was characterized by poor LASV-specific T cell responses [68], suggesting an inability of the host to control virus infection. In addition, elevated serum levels of plasminogen activator inhibitor-1, soluble thrombomodulin, and tumor necrosis factor receptor 1 were also correlated with fatal LF [69], implicating endothelial dysfunction and coagulopathy in disease severity. The contribution of viral factors to disease severity has also been demonstrated in animal model studies. ML29 is a reassortant virus containing the S segment of LASV Josiah strain and the L segment of Mopeia virus (MOPV). Studies with ML29 indicate that the LASV L segment was critical for maintaining virulence in animal models [70]. Consistent with this finding, reassortment studies using a lethal LASV strain (LF2384) and a nonlethal strain (LF2350) identified the L protein encoded by the LF2384 L segment as a major determinant of high mortality in guinea pigs [12]. A LASV reassortant study involving the Josiah strain and a more recently isolated 2015 Liberian strain indicated the S segment of the LASV Josiah strain was sufficient to maintain pathogenicity in guinea pigs [71]. At the viral protein level, LASV NP contains a conserved exoribonuclease (ExoN) motif, which mediates the degradation of virus-derived, immunostimulatory double-stranded RNAs (dsRNAs) generated during viral replication and facilitates LASV immune evasion [72,73,74]. Further studies indicate that LASV L protein collaborates with NP to restrict dsRNA accumulation, which is a unique characteristic of LASV host immune evasion [75]. In addition, LASV Z protein inhibits interferon production and may also contribute to viral pathogenicity [51]. Collectively, these data suggest that multiple viral factors can affect LASV virulence and vary according to virus strain and experimental model.

## 5. Host Immune Response to LASV Infection

Clinical and animal studies have demonstrated the critical role of host immune responses in LASV pathogenesis and disease outcome (reviewed in [76,77,78,79,80,81,82,83,84,85,86]). Severe LASV infection is typically characterized by high levels of viremia, weak and late interferon (IFN) and cytokine responses, as well as impaired activation of dendritic cells, macrophages, and LASV-specific T lymphocytes [78,87]. In contrast, early and robust immune responses, particularly LASV-specific T cell responses, correlate with viral clearance and host survival [76,81,88].

### 5.1. LASV Evasion of the Innate Immune Response

Severe LASV infection is associated with suppression of innate immune responses. Upon RNA virus infection, host pathogen pattern recognition receptors (PRRs), such as retinoic acid-inducible gene I (RIG-I) and Melanoma Differentiation-Associated Protein 5 (MDA5), can sense non-self-viral RNAs, which typically contain double-stranded RNA structures [76,89,90]. Activation of PRRs initiates a cascade of downstream signaling, leading to activation of interferon regulatory factors (IRF3 and IRF7) and nuclear factor kappa B (NF-κB) [76] followed by the expression of type I IFN α and β. Produced Type I IFNs are secreted from infected cells, bind to the IFN receptors on the cell surface in an autocrine or paracrine manner, and further induce the expression of various IFN-stimulated genes (ISGs). ISGs execute IFN-induced antiviral activities by targeting various steps of the viral life cycle. Expression of a subset of ISGs can be directly mediated by IRF-3/IRF-7 in an IFN-independent manner, particularly in dendritic cells and macrophages.

The mechanism underlying LASV suppression of innate immune responses has been studied extensively. LASV is known to limit accumulation of immune-stimulatory dsRNAs in infected cells [75]. Moreover, NP and Z proteins suppress type I IFN and cytokine expression by inhibiting the RIG-I-like receptor (RLR) signaling pathway and the protein kinase R (PKR) pathway [72,75,76,89,91,92,93,94]. Mechanistically, LASV NP directly interacts with cellular sensors of viral RNA and downstream signaling factors, including RIG-I, IκB kinase epsilon (IKKε), and mitochondrial antiviral signaling protein (MAVS) [72,73,95,96]. LASV Z directly interacts with and inhibits RLR [51,97]. The precise contribution of NP- and Z-mediated IFN suppression to LASV pathogenicity remains to be fully defined. More detailed discussions on LASV suppression of host innate immune responses have been reviewed elsewhere [76,79,80].

### 5.2. LASV Interference with Antigen Presentation

In the early stage of infection in vivo, LASV primarily targets host macrophages and dendritic cells, which also mediate host innate and adaptive immune responses. Studies with cynomolgus monkeys indicated that macrophages and dendritic cells facilitate LASV (Josiah and AV strains) dissemination to draining lymph nodes and promote systemic spread [36,98]. In *M. natalensis*, macrophages and dendritic cells were permissive to LASV infection. However, infection with LASV Josiah strain did not induce the expression of antiviral or maturation-associated genes [99], and the Ba366 strain triggered only weak IFN responses [100]. Similarly, LASV (AV strain) could efficiently replicate in human monocyte-derived dendritic cells (moDCs) and macrophages ex vivo, but these cells neither activated nor produced proinflammatory cytokines [101]. Human moDCs infected by LASV Niahun strain also failed to activate T lymphocytes [102]. In LASV AV strain infection, a modest type I IFN response was observed in human macrophages, but not in moDCs [103]. Another study further found that although human macrophages infected by LASV (AV strain) were able to activate natural killer (NK) cells, these NK cells failed to suppress virus infection [104]. Myeloid dendritic cells (mDCs) are specialized in antigen presentation and induction of T cell responses. Human mDCs infected by LASV AV strain exhibited an impaired capacity to activate T lymphocytes in an mDC and autologous T cell co-culture system [105]. These studies demonstrated that LASV efficiently interferes with the functions of host antigen-presenting cells.

### 5.3. Adaptive Immune Response in LASV Infection

The role of T cell responses in human infection remains poorly understood due to the scarcity of immunological data from patients. In a patient who recovered from acute LF, a robust CD8^+^ T cell response was observed concomitantly with viremia decline [42]. Severe LF cases were associated with poor LASV-specific effector T cell responses along with the expansion of LASV non-specific T cells with homing capacity to inflamed tissues [68]. In cynomolgus monkeys, lethal LASV infection with the Josiah strain was characterized by an expansion of myeloid suppressor cells and suppression of LASV-specific T cell responses. In contrast, control of non-lethal infection with the LASV AV strain was associated with induction of an LASV-specific T cell response [106]. These human and clinical data support a model that LASV clearance and host recovery is correlated with effective LASV-specific T cell responses, preferably at the early stage of infection, while severe and fatal LASV infection is associated with suppressed LASV-specific T cell responses as well as non-specific bystander T cell responses that contribute to LF immunopathology. Due to lack of clinical data, the role of T cell responses in determining differential outcomes in human infection, and importantly the underlying mechanism, remains to be defined. Systematic and rigorous studies in human patients are necessary to elucidate the pathogenesis of LF, which may also inform the development of effective therapeutics and countermeasures. In addition, durable LASV-specific T cell responses were detected for 1–2 years in LF survivors [107] and some LF survivors developed inter-lineage, cross-reactive T cell responses [107,108]. These findings underscore the importance of eliciting LASV-specific T cell responses as a key strategy for the development of broadly protective LASV vaccines.

There is accumulating evidence indicating a critical role of T cell-mediated immune responses in LASV-induced neurological sequelae. Approximately one-third of LF survivors develop an often bilateral and permanent SNHL in the late stages of acute disease or in the early convalescent phase [109,110]. The cause of the high incidence of SNHL in LF patients remains to be determined. Data from animal model studies suggest that SNHL is more likely driven by host immune responses than by direct virus-mediated damage [110,111]. In STAT1 knockout mice but not in IFNα/βγ receptor knockout mice, infection with LASV LF2350 strain caused hearing loss resembling the SNHL in LF patients [111]. While LASV antigens could be detected in the inner ears of both types of mice, tissue damage was only observed in the STAT1 knockout mice, concomitant with profound CD3-positive lymphocytic infiltration [111,112]. Moreover, depletion of CD4+ T cells prevented the infected STAT1 knockout mice from developing hearing loss, indicating the essential role of CD4+ T cells in LASV-induced SNHL [113].

Humoral responses do not correlate with the survival or viral clearance in LF patients [114]. LASV neutralizing antibodies are usually low or not detected in the late or convalescence stage of LASV infection [76,77,78,107,115]. B cell responses appeared to be suppressed in guinea pigs infected with clinical LASV isolates [12]. Nevertheless, neutralizing antibodies from survivors showed cross-lineage reactivity and could last for six months or longer after recovery [107,114,115]. Importantly, studies on survivor-derived monoclonal antibodies and vaccine-elicited antibody responses indicated that neutralizing antibodies could confer protection [116,117,118], underscoring their importance in therapeutic and vaccine development.

### 5.4. Cytokine and Chemokine Response as Biomarkers of LASV Infection

Cytokine and chemokine responses have also been assessed as biomarkers of LF. Branco L et al. reported a correlation of lower levels of interleukin (IL)-6, -8, -10, and other cytokines with the survival of LF patients [119]. Consistently, Strampe J et al. found a correlation of upregulated levels of IL-6, IL-8, and IP-10 with fatal LF outcomes [69]. However, Mahanty S et al. observed low or undetectable levels of IL-8 and IP-10 in patients with fatal LF [120]. It remains to be addressed whether this discrepancy may be attributed to inclusion of human cohorts with frequent exposure to parasitic infections or to other factors [119].

## 6. Medical Countermeasures

Although LASV poses a substantial public health threat in endemic countries and among travelers, there are no approved countermeasures or vaccines [114]. The sequences of LASV genomic RNA are highly diverse among different strains. The nucleotide sequence difference can be as high as 25% and 32% in S and L genomic RNA segments, respectively. New LASV lineages have been identified in the past decades, and additional lineages are likely to be detected with continued surveillance. The genetic diversity of LASV poses a challenge to the development of broad-spectrum antivirals, vaccines, and diagnostics. Historically, experimental infection studies as well as the evaluation of antivirals and vaccines have largely relied on the Josiah strain of LASV, which was isolated 50 years ago and may not fully represent LASV strains circulating in the field. Therefore, isolation and characterization of contemporary LASV strains from patients and natural hosts is essential for LASV surveillance and the evaluation of medical countermeasures in pre-clinical and clinical studies.

### 6.1. Antivirals

Clinical treatments primarily rely on supportive care and the off-label use of ribavirin [121,122]. Historical clinical observation data suggested a possible greater benefit when administrated earlier. However, the clinical evidence has important methodological limitations, and the historical evidence base has undergone substantial re-evaluation [123]. The magnitude of the benefit and the optimal treatment window remain uncertain. In addition, experimental infection in mouse models suggests that ribavirin may reduce inflammation-associated cell death [124].

To date, antiviral development has mainly focused on inhibiting viral RNA replication or virus entry. Favipiravir, a purine analogue, was originally developed as an influenza virus inhibitor. A combination of ribavirin and favipiravir was used for treatment in two LF cases [42,125]. Both patients survived but the contribution of favipiravir remained to be defined. Favipiravir was subsequently evaluated in outbred guinea pigs and demonstrated protection efficacy against lethal infection with the guinea pig-adapted LASV Josiah strain [126]. Further evaluation in macaques infected with a lethal dose of LASV Josiah strain indicated that successful treatment in NHPs required a high dose of favipiravir (300 mg/kg/day) [127]. Due to its teratogenic and embryotoxic effects, favipiravir is restricted for use in pregnancy.

4′-fluorouridine (4′-FlU) is an orally available ribonucleotide analogue. It demonstrated potent antiviral efficacy against lethal infection with the LASV Togo strain (lineage VII) in NHPs [128] and the LASV Josiah strain (lineage IV) in inbred guinea pigs [129]. 4′-FlU acts by inhibiting LASV RNA polymerase and shows potent efficacy (EC50: 1.0–10.08 μM) against a broad spectrum of LASV lineages (I−IV and VI−VII) in Hela cells and Huh7 cells [129]. Notably, the antiviral exhibited therapeutic efficacy at low doses in animals (0.5 mg/kg) and was effective at 6 or 7 days post-exposure [128,129]. Despite its promising efficacy in preclinical studies, the safety and efficacy of 4′-FlU in humans remain to be evaluated in clinical trials.

LASV entry inhibitor LHF-535 demonstrated potent efficacy in pre-clinical studies and entered a Phase 1 clinical trial. Treatment with LHF-535 (50 mg/kg/day) at day 1 after infection fully protected inbred guinea pigs from lethal challenge with guinea pig-adapted LASV Josiah strain and was effective at 3 days after infection [130]. In June 2020, LHF-535 finished a Phase 1 clinical trial (ClinicalTrials.gov ID NCT03993704) [131] and was well tolerated in participants, demonstrating rapid absorption and a long half-life [132]. In addition to LASV, LHF-535 exhibited antiviral efficacy against other arenaviruses including Tacaribe virus (TCRV). Notably, serial passage of TCRV in LHF-535-treated Vero cells yielded drug-resistant TCRV with GP2 mutations [133]. The majority of LHF-535-resistant TCRV mutants were found to be attenuated in an AG129 mouse model [133]. The emergence of drug-resistant mutations in a related arenavirus underscores the importance of assessing potential resistant LASV mutants during the development of LHF-535 as a therapeutic for LF.

Orally bioavailable ARN-75039 has shown strong protective efficacy against lethal infection with a guinea pig-adapted LASV (Josiah) in outbred guinea pigs [134]. In the study, ARN-75039 treatment started either 3 days after LASV infection in a once-daily regimen or 7 days after infection in a twice-daily regimen for 14 days. Both drug regimens achieved 100% protection at the doses of 3.75 mg/kg (once-daily administration) or 7.5 mg/kg (twice-daily administration). In the group treated with 15 mg/kg ARN-75039 3 days after infection, one animal (1/10) succumbed. Among the animals treated with ARN-75039 7 days after infection, one animal (1/10) in the 15 mg/kg group and two animals (2/10) in the 30 mg/kg group died. In March 2023, ARN-75039 completed a Phase 1 clinical trial for safety, tolerability, and pharmacokinetic study (ClinicalTrials.gov ID NCT05735249) [135]. In February 2026, ARN-75039 advanced to Phase 2 clinical evaluation for the safety, tolerability, and treatment efficacy in LF patients (ClinicalTrials.gov ID NCT07419373) [136]. The trial was subsequently placed on a full clinical hold by the FDA in June 2026 and the basis for the hold has not been publicly reported.

### 6.2. Vaccines

There is an urgent need for effective and safe LASV vaccines that provide broad protection against different LASV lineages and are cost-effective. LASV vaccine development needs to consider both the immune mechanisms targeted for protection and the viral antigens selected to elicit those responses. Because LASV-specific T cell responses have been widely recognized as a key correlate of protection in LASV infection, induction of T cell immunity is a reasonable strategy for vaccine development. In addition, accumulating evidence indicates that antibody responses, primarily neutralizing antibodies (nAbs), contribute to vaccine-mediated protection. Consequently, induction of both robust cellular and humoral immune responses should be considered to achieve optimal protective efficacy of LASV vaccines. Among LASV proteins, GPC is the primary target for vaccine development because it is the sole viral surface protein and the primary target of protective nAbs. The NP is the most conserved and abundant protein in virions and infected cells and is known to induce robust cross-reactive T cells and non-nAbs. Therefore, the NP has also been incorporated into some vaccine platforms. The first experimental vaccine, vaccinia virus expressing LASV NP, fully protected guinea pigs against fatal LF [137]. Accordingly, an optimal LASV vaccine strategy may consider co-expression of NP and GPC antigens to elicit robust humoral and cellular response and provide broad protection. In addition to efficacy and safety, vaccine affordability is also a key factor to be considered. A recent health-economic analysis estimated that the price of a cost-effective LASV vaccine should be lower than $10 per dose [138].

Several LASV vaccine candidates have demonstrated promising preclinical results and progressed to clinical trials (summarized in a recent review [114]). The vaccine candidates that have entered or completed clinical trials are summarized in Table 2. The rVSVΔG-LASV-GPC, which utilizes the recombinant vesicular stomatitis virus expressing LASV GPC (Josiah, lineage IV), completed Phase 1 clinical trials in March 2024 (ClinicalTrials.gov ID NCT04794218) [139], showing no serious vaccine-related adverse events including hearing loss [118]. This vaccine candidate induced durable humoral and T cell immune responses towards common LASV lineages in humans [118], including robust binding antibody responses against LASV lineages I−IV and VII, nAbs against LASV lineages II−IV, and LASV-specific T cell responses that lasted for at least one year. Since March 2024, a Phase 2 clinical trial (ClinicalTrials.gov ID NCT05868733) has been started in West Africa to evaluate the safety, tolerability, and immunogenicity of the rVSV∆G-LASV-GPC vaccine in adults and children [140]. The MV-LASV vaccine candidate [141,142,143], which employs a measles virus vector expressing both GPC and NP antigens, elicited substantial LASV-specific IgG responses and demonstrated an acceptable safety and tolerability profile in a Phase 1 clinical trial (ClinicalTrials.gov ID NCT04055454). However, nAb and LASV-specific T cell responses were not detected in the trial [144]. The DNA vaccine INO-4500, which expresses LASV GPC, finished Phase 1a and 1b clinical trials in October 2022 (ClinicalTrials.gov ID NCT03805984 and NCT04093076) [145]. The vaccine candidate was well tolerated and induced LASV-specific T cell responses in 86% of participants at week 12 in the high-dose group. However, the study did not evaluate the nAb response and reported a low seroconversion rate of the binding antibody (<10%) through week 12 [146]. An inactivated rabies virus-vectored LASV GPC vaccine (LASSARAB) has started Phase 1 clinical trials in January 2025 (ClinicalTrials.gov ID NCT06546709) [147], and was found to be well tolerated in participants and achieved a seroconversion rate of 100% [148]. The nAb and T cell responses were not evaluated in the Phase 1 trial as these responses were not associated with protection in LASSARAB preclinical studies.

### 6.3. Diagnostics

Diagnosis of LASV infection is critical for effective LF management and reducing the risk of nosocomial transmission. However, early and prompt diagnosis of LF is challenging in clinical settings due to the non-specific, early-stage LF disease signs and symptoms resembling other infectious diseases that are endemic in the same regions [87]. Delayed and missed diagnoses increase the risk of hospital-acquired LASV infection among healthcare workers and patients. There is an urgent need for rapid and reliable diagnostic tools suitable for point-of-care use. Current LASV diagnostics utilize a wide range of techniques such as PCR, ELISA, CRISPR diagnostics, isothermal amplification assays, and biosensor platforms to detect viral RNA, antigens, and antibodies (refer to [149] for a more comprehensive review). Among them, real-time reverse transcription PCR (RT-PCR) detection of LASV RNA is the most widely used laboratory method for LF diagnosis [150]. The high sequence diversity of LASV presents a major obstacle to the design of primers and probes for the detection of genetically diverse LASV isolates with high sensitivity and specificity. As of October 2025, there are twenty-four commercial LASV tests on the market and four of the molecular tests have regulatory approval [149]. The Lassa Virus (LV) Real Time RT PCR Kit (Liferiver Bio-Tech, US Corp/Shanghai ZJ Bio-Tech Co, La Jolla, CA USA), certified for clinical diagnostic use in the European Union, can detect major LASV lineages (I−VI) in laboratory settings. The assay exhibits an analytical sensitivity of 1000 viral RNA copies/mL of serum. However, real-time RT-PCR requires specialized infrastructure and trained personnel, which may not be readily available in rural healthcare settings. BioFire Defense LLC (Salt Lake City, UT, USA) has developed an FDA-authorized, near-point-to-care assay: Global Fever Special Pathogens Panel. This is a qualitative, multiplexed, nucleic acid-based test enabling simultaneous detection of multiple viral, bacterial, and parasitic pathogens, including LASV, Ebola virus, Marburg virus, Crimean-Congo hemorrhagic fever virus, and Dengue virus. The assay exhibits an analytical sensitivity of 5600 viral RNA copies/mL of whole blood for LASV Josiah strain (lineage IV). The assay’s diagnostic performance for other lineages or circulating LASV strains is not documented.

Efforts to improve rapid LF diagnosis have also focused on point-of-care tests for LASV antigen. Among them, the ReLASV Pan-Lassa Antigen Rapid Test (Pan-Lassa RDT, Zalgen Labs, Frederick, MD, USA) was developed to reduce dependence on high-technology laboratories and lower diagnostic turnaround time [151]. However, the Pan-Lassa RDT has been discontinued due to its diagnostic performance. In a field trial involving 217 participants at a federal tertiary hospital in Abakaliki, Nigeria, the Pan-Lassa RDT demonstrated a low sensitivity (<10%) [152]. In another field trial in Nigeria, the assay exhibited a low sensitivity (65.0%) and specificity (50.7%) [153]. A rapid diagnostic test has been developed using newly identified monoclonal antibodies, which exhibits higher sensitivity than the Pan-Lassa RDT and broad lineage coverage [154]. Despite the development efforts and recent progress, rapid and reliable LASV diagnostics for non-laboratory use remain a critically unmet need.

## 7. Conclusions, Important Gaps, and Future Directions

LASV continues to pose a persistent threat to public health in West Africa and may expand to other regions due to increasing international travel. Despite its importance, the true disease burden in endemic regions is still not well understood, underscoring the need for continued and enhanced surveillance efforts to inform disease control. Research into LASV pathogenicity and host immune responses in relevant animal models has advanced our understanding of the viral and host factors that are critical for LASV infection and disease outcomes. However, critical gaps remain in our understanding of immune response, viral pathogenesis, and neurological sequelae in human infections, particularly the humoral and cellular immune correlates of protection and disease severity, as well as the mechanisms driving hearing loss and other long-term complications. Investigating LASV infection in immune-privileged tissues, such as CNS and reproductive organs, is critical to understanding viral persistence and pathogenesis. Addressing these knowledge gaps would inform the development of effective LF therapies and vaccines.

Despite significant advances in the development of medical countermeasures, no safe and broadly protective antiviral or vaccine has yet been approved for clinical use. Accumulating evidence indicates a substantial rate of LASV infection in children, highlighting the importance to consider pediatric populations in vaccination strategy. Vaccine development should also consider the cost-effectiveness. In addition, there is an unmet urgent need for rapid and reliable diagnostic tools suitable for point-of-care use, which are essential for timely diagnosis and treatment of LF patients to reduce disease burden in endemic areas and mitigate the potential spread of LASV. Given the high sequence diversity of LASV, continued efforts in characterizing the sequence of circulating LASV strains would be critical for the development of broad-spectrum diagnostics and LASV surveillance. Successful vaccines and antivirals should be effective against circulating LASV strains. Notably, vaccine and antiviral development have largely relied on the Josiah strain (lineage IV), which may not fully represent circulating LASV strains. Isolation of contemporary LASV strains would therefore provide valuable tools essential for assessing the efficacy of candidate vaccines and antivirals against circulating viruses.

As LF is a zoonotic disease driven by interactions among rodent reservoir, ecological changes, and human behavior, effective control requires coordinated actions across public health, animal health, and community sectors. A comprehensive One Health strategy that integrates human, animal, and environmental health interventions is essential to ensure timely and effective surveillance, prevention, and outbreak preparedness.

## Figures and Tables

**Table 1:** Lassa fever cases reported in Nigeria (2018–2026).

Year	Suspected Cases	Confirmed Cases	Deaths	Case Death Rate
2018	3498	633	171	27%
2019	5057	833	174	21%
2020	6791	1189	244	21%
2021	4654	511	102	20%
2022	8202	1067	189	18%
2023	9155	1270	227	18%
2024	10,098	1309	214	16%
2025	9389	1148	215	19%
2026 *	5652	855	214	25%
Total	62,496	8815	1750	20%
Average/Year **	7106	995	192	19%

2026 *: by week 23; Average/Year **: annual average between 2018 and 2025. Data source: Nigeria CDC [13].

**Table 2:** LASV vaccine candidates that have entered or finished clinical trials.

Candidate	Platform	Antigen	Clinical Stage	Main Findings
rVSVΔG-LASV-GPC	Replication-competent recombinant VSV	GPC (Josiah)	Phase 1 (NCT04794218): completed (March 2024); Phase 2 (NCT05868733): started from March 2024	Acceptable safety profile; no vaccine-related serious adverse events; durable cellular immunity, binding antibodies, and neutralizing antibodies with cross-reactive responses to multiple LASV lineages
MV-LASV	Live recombinant measles virus vector	GPC and NP (Josiah)	Phase 1 (NCT04055454): completed (15 January 2021)	Acceptable safety and tolerability profile; potent LASV-specific IgG; no measurable nAb and LASV-specific T cell responses
INO-4500	DNA vaccine	GPC (Josiah)	Phase 1a (NCT03805984): completed (21 October 2020) Phase 1b (NCT04093076): completed (14 October 2022)	Acceptable tolerability profile; LASV-specific T cell responses; low seroconversion rate (<10%).
LASSARAB	Inactivated rabies virus-vectored	GPC (Josiah)	Phase 1 (NCT06546709): started (13 January 2025)	Acceptable safety profile; 100% IgG seroconversion; nAb and T cell responses not evaluated

## Data Availability

The original contributions presented in this study are included in the article/supplementary material. Further inquiries can be directed to the corresponding author.

## Supplementary Materials

The following supporting information can be downloaded at https://www.mdpi.com/article/10.3390/microorganisms14092047/s1, The Supplementary Dataset for human LASV isolates.
